# Supplementary material for: Spatial Variation in Stream-Water Bacterial Communities Across Sites Differing in Wild Chinese Giant Salamander Detection Frequency
Source: Biology (Basel). 2026 Sep 18;15(18):1647. doi: 10.3390/biology15181647 (PMC13604818; doi:10.3390/biology15181647)
Supplement: Supplementary file 1 [file biology-15-01647-s001.zip › Data Related to “Spatial Variation in Stream-Water Bacterial Communities across Sites Differing in Wild Chinese Giant Salamander Detection Frequency”/Microbial Data/result/03.TaxonVisual/heatmap/sample/heatmap.sample.o.pdf]

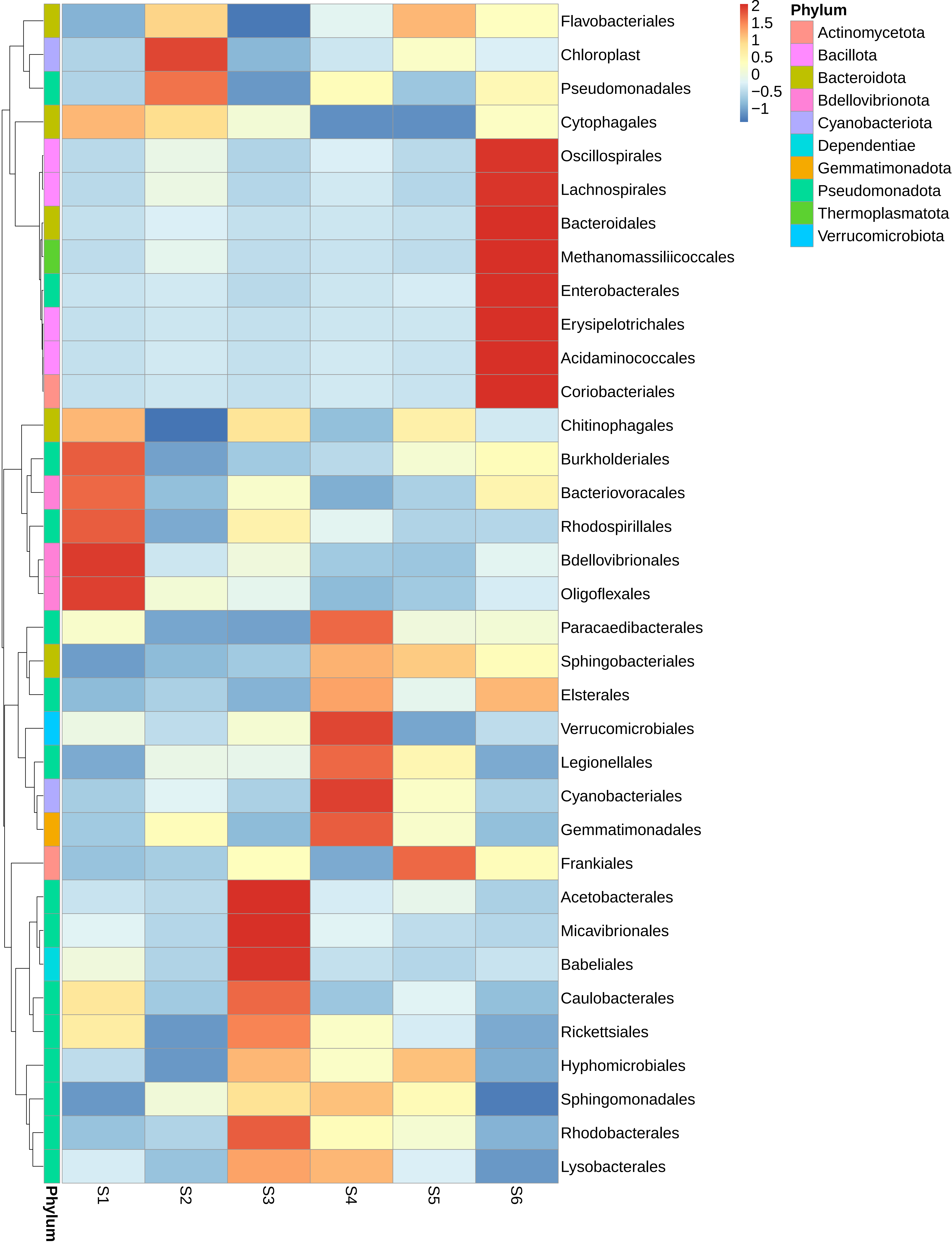
Flavobacteriales
Chloroplast
Pseudomonadales
Cytophagales
Oscillospirales
Lachnospirales
Bacteroidales
Methanomassiliicoccales
Enterobacterales
Erysipelotrichales
Acidaminococcales
Coriobacteriales
Chitinophagales
Burkholderiales
Bacteriovoracales
Rhodospirillales
Bdellovibrionales
Oligoflexales
Paracaedibacterales
Sphingobacteriales
Elsterales
Verrucomicrobiales
Legionellales
Cyanobacteriales
Gemmatimonadales
Frankiales
Acetobacterales
Micavibrionales
Babeliales
Caulobacterales
Rickettsiales
Hyphomicrobiales
Sphingomonadales
Rhodobacterales
Lysobacterales
S1
S2
S3
S4
S5
S6
Phylum
2
1.5
1
0.5
0
−0.5
−1
Phylum
Actinomycetota
Bacillota
Bacteroidota
Bdellovibrionota
Cyanobacteriota
Dependentiae
Gemmatimonadota
Pseudomonadota
Thermoplasmatota
Verrucomicrobiota
